# Carbohydrates for Soccer: A Focus on Skilled Actions and Half-Time Practices

**DOI:** 10.3390/nu10010022

**Published:** 2017-12-25

**Authors:** Samuel P. Hills, Mark Russell

**Affiliations:** School of Social and Health Sciences, Leeds Trinity University, Horsforth, Leeds LS18 5HD, UK; 1705952@leedstrinity.ac.uk

**Keywords:** glycemia, football, skill, ergogenic, blood glucose, cognition

## Abstract

Carbohydrate consumption is synonymous with soccer performance due to the established effects on endogenous energy store preservation, and physical capacity maintenance. For performance-enhancement purposes, exogenous energy consumption (in the form of drinks, bars, gels and snacks) is recommended on match-day; specifically, before and during match-play. Akin to the demands of soccer, limited opportunities exist to consume carbohydrates outside of scheduled breaks in competition, such as at half-time. The link between cognitive function and blood glucose availability suggests that carbohydrates may influence decision-making and technical proficiency (e.g., soccer skills). However, relatively few reviews have focused on technical, as opposed to physical, performance while also addressing the practicalities associated with carbohydrate consumption when limited in-play feeding opportunities exist. Transient physiological responses associated with reductions in activity prevalent in scheduled intra-match breaks (e.g., half-time) likely have important consequences for practitioners aiming to optimize match-day performance. Accordingly, this review evaluated novel developments in soccer literature regarding (1) the ergogenic properties of carbohydrates for skill performance; and (2) novel considerations concerning exogenous energy provision during half-time. Recommendations are made to modify half-time practices in an aim to enhance subsequent performance. Viable future research opportunities exist regarding a deeper insight into carbohydrate provision on match-day.

## 1. Introduction

Soccer is the world’s most popular sport [1], and is typically contested over two 45-min halves, each separated by a ~15 min half-time break. A literature-wide consensus exists that modern day players cover 10–12 km over the full 90 min [2,3,4,5,6,7,8]. While the predominant activities in soccer are of low-intensity, and primarily aerobic in nature [9,10,11], the importance of anaerobic metabolism is highlighted by observations that over 300 accelerations and decelerations may occur during each half [2], with a sprint being performed every ~90 s [3]. Despite high-intensity distance making up only 1–11% of the total distance covered during a match [3], the most decisive passages of play typically involve high-intensity actions, such as sprinting or the execution of game-specific skills [4]. Additionally, as the primary objective of soccer is to score more goals than the opposition, full-match players complete over 100 technical or skilled involvements [12]. An average of 10 shots and between 16–30 attacking plays are required for each goal scored; with dribbling and short passing being the most frequently performed technical actions [12,13]. Accordingly, interventions that improve skilled as well as physical actions are likely of interest to practitioners responsible for enhancing soccer performance.

The importance of carbohydrates in soccer has been acknowledged since the early 1970’s. Seminal work interrogating muscle biopsy data, identified compromised muscle glycogen stores following soccer match-play, and observed concomitant declines in physical performance [14]. Players beginning the game with higher muscle glycogen stores (~400 mmol kg^−1^ dry weight; d.w) achieved higher movement intensities, and were better able to maintain total distance covered between-halves, than those who began with reserves of ~200 mmol kg^−1^ d.w [14]. Interestingly, Krustrup et al. [11] identified that almost 50% of individual fibers were empty, or almost empty, after 90 min of intermittent exercise; a response which was proposed to undermine force-production capabilities, even if total muscle glycogen was not substantially reduced. Given the performance benefits of attenuating glycogen depletion throughout a match, it is unsurprising that soccer players are recommended to consume 30–60 g·h^−1^ of exogenous carbohydrate during competition to maintain blood glucose concentrations and spare muscle glycogen reserves [15]. Accordingly, a 22% reduction in muscle glycogen utilization has been observed when players ingested ~1.1 L of a 6.9% carbohydrate solution throughout a simulated soccer match [16]. 

While the ergogenic effects of carbohydrate ingestion have been confirmed in relation to soccer performance (for reviews, see: [17,18]), studies have traditionally focused on physical actions performed during protocols, often executed on motorized treadmills (for example: [19]), that have frequently overlooked potentially important facets of the game. In a study incorporating neither skilled actions nor a scheduled half-time break, Nicholas et al. [20] observed that consuming a 6.9% carbohydrate–electrolyte drink during 75 min of intermittent exercise, increased time to exhaustion during a subsequent running assessment. Notwithstanding the importance of maintaining physical performance, execution of soccer-specific technical skills may be a crucial determinant in the outcome of a game [21]. This narrative review summarizes pertinent research that evaluates the efficacy of carbohydrates on soccer-specific technical performance. Computerized literature searches were performed in PubMed, Google Scholar and SportDiscus databases between April 2017 and November 2017. Keywords relating to the sport (e.g., ‘soccer’, ‘football’), outcomes of interest (e.g., ‘skill’, ‘technical’, ‘passing’, ‘shooting’, ‘dribbling’, ‘juggling’), interventions (e.g., ‘carbohydrate’, ‘glucose’, ‘maltodextrin’, ‘isomaltulose’, ‘maltose’, ‘sucrose’, ‘glycemic index’) and other key terms (e.g., ‘half time’, ‘blood glucose’, ‘glycogen’) were used in different combinations. Articles evaluating technical proficiency in ‘rugby’ were excluded. All titles were scanned and relevant articles were retrieved for review. In addition, the reference lists from both original and review articles retrieved were also reviewed. Evidence highlights novel considerations for match-day carbohydrate consumption by soccer players, namely: (1) A role in the maintenance of skilled performance; and (2) possible modification of carbohydrate feeding strategies throughout match-play (with an emphasis on scheduled breaks in play, i.e., half-time).

## 2. Carbohydrates and Skilled Actions Performed during Soccer-Specific Exercise

Considering the disproportionate number of goals scored from 75 min onwards during soccer matches [22], and the acknowledged importance of key skills such as passing and shooting [23,24], the ability of a team to maintain technical proficiency while engaged in prolonged intermittent exercise is likely a key determinant of competitive success. Indeed, as exercise progresses into the second half, and potentially extra-time, important aspects pertaining to passing and shooting performances deteriorate [25,26]. Although the mechanisms underpinning these effects remain unclear, and environmental or match-specific factors may contribute, reductions in fuel availability (i.e., depressed muscle glycogen and blood glucose concentrations [8,10,11]), impaired cognitive function (i.e., reduced decision making skills and increased reaction times [27,28,29]), and dehydration (i.e., fluid losses in excess of 2% body mass, BM, increasing thermal strain [8,30]) have been implicated. It appears likely that the progressive declines in skilled performance observed during soccer matches are multifactorial in origin.

Over the past decade, research into the efficacy of nutritional ergogenic aids for soccer skills performed during fatiguing exercise has accumulated. The most common approaches have revolved around the use of carbohydrates; although the ergogenic effects of caffeine and fluid provision have also been reported [18]. In a systematic review of the literature, 75% of eligible carbohydrate papers identified that 6–8% solutions of glucose, sucrose or maltodextrin, ingested at a rate of 30–60 g·h^−1^, enhanced at least one aspect of skilled performance over the duration of soccer-specific exercise [18].

As the brain is one of the few human organs reliant primarily on blood glucose for optimal functioning [31], it is probable that decision-making and the performance of skilled actions during soccer match-play are influenced by blood glucose concentrations. Evidence from non-exercise studies highlights that cerebral glucose uptake begins to decline when blood glucose concentrations fall below 3.6 mmol∙L^−1^ [32]; a level which, although rare, is similar to those previously reported in soccer players [33]. Indeed, the relationship between glucose availability and cognitive performance has been confirmed in both healthy and diabetic populations [29,34,35,36]. In respect to the present discussion, Bandelow et al. [28] observed faster visual discrimination, fine motor speed, and psycho-motor speed in participants with increased blood glucose concentrations following soccer match-play in hot conditions. As cognitive processes are crucial to the skilled actions involved in team sports, and considering the role of blood glucose in the maintenance of brain function, supplementing exogenous carbohydrates that influence blood glucose concentrations could be of benefit to soccer skills performed in the latter stages of a match.

Ergogenic effects have been identified when exogenous carbohydrates are ingested before and throughout 90 [26], and 120 [25] min of soccer-specific activity. Specifically, a 6% sucrose–electrolyte drink consumed throughout exercise improved the speed of shots taken by professional youth soccer players during the second half of a soccer match simulation, whilst allowing accuracy to be maintained [26]. Similarly, carbohydrate gels (2 sachets, each containing 23 g of glucose and maltodextrin) provided to professional English Premier League youth soccer players before a simulated extra-time period, improved dribbling performance during the final 15 min of extra-time, compared with an energy-free placebo [25].

While evidence suggests that provision of exogenous carbohydrates may represent an acute strategy to prevent reductions in skilled performances throughout soccer-specific exercise, practitioners must consider the logistical implications of achieving ergogenic rates of intake (e.g., >50 g·h^−1^ [37]), without compromising gastric tolerance [38]. Although many studies have provided carbohydrates at regular intervals during exercise (i.e., every ~15 min; [26,39]), the limited number of stoppages in soccer match-play means that the potential for such frequent feeding may not exist. Indeed, the combination of high sweating rates and sporadic opportunities to drink can impair maintenance of fluid balance in soccer [40,41]; with empirical observations identifying that the warm-up and half-time are key opportunities to ingest carbohydrates. Although comparable metabolic responses (i.e., blood glucose, carbohydrate and fat oxidation) were observed when equivalent amounts of carbohydrate (~68 g ingested at ~45 g·h^−1^) were consumed in two (i.e., before each half) or six (i.e., every 15 min) boluses, the volume of fluid ingested per feeding (>500 mL) in the two bolus trial meant that gut fullness ratings were elevated versus the high-frequency ingestion trial [42]. 

Accordingly, when limited opportunities exist to drink before and during match-play, consuming electrolyte beverages that contain increased (>10%) carbohydrate concentrations may provide a practical approach to enable soccer players to achieve the desired energy intake, whilst minimizing abdominal discomfort. Harper et al. [43] reported that a 12% carbohydrate–electrolyte solution, delivered in 250 mL boluses prior to the beginning of each half, improved dribbling speed and self-paced exercise performance during the later stages of a soccer match simulation, compared with either water or an electrolyte placebo. Crucially, the similar gut fullness ratings between the three conditions suggest in favor of consuming higher-concentration carbohydrate–electrolyte beverages as a practical strategy to meet the ergogenic threshold of carbohydrate ingestion, which may help attenuate declines in technical and physical performance throughout a game.

It is also important to note that the performance of skilled tasks may also be influenced by playing experience and/or physical maturity. Accordingly, it may therefore be of interest to consider whether the effects of match-day consumption of carbohydrates are the same for youth or adolescent soccer players as they are in older populations. While unrelated to soccer, improvements in basketball shooting accuracy and on-court sprinting performance have been observed when 12–15 years old players remained euhydrated with a 6% carbohydrate–electrolyte solution versus water consumption during exercise [44]. Conversely, neither shooting accuracy nor the sprinting performance of 14–15 years old basketball players were influenced by ad libitum consumption of an 8% carbohydrate–electrolyte solution compared with water [45]. As limited and equivocal literature exists on the efficacy of carbohydrate supplementation in adolescent athletes, particularly with reference to skilled actions in soccer, future research opportunities exist in this area. 

## 3. Modification of Half-Time Practices

### 3.1. Current Half-Time Practice

In soccer, consecutive halves are separated by a scheduled 15 min pause in competition. This interlude may be considered a period of recovery following the first half, as well as an opportunity to prepare for the subsequent passage of play. Empirical and published evidence alike [46], highlights that upon returning to the changing room following cessation of the first half, players primarily engage with tactical debriefing alongside medical and/or nutritional practices; which include the consumption of carbohydrate–electrolyte beverages and high glycemic index (GI) foods. Personal preparation, addressing playing kit/equipment concerns, receiving video feedback, and individual player/coach interactions may also follow. Therefore, apart from potentially a short period of rewarm-up activity (which all clubs would not universally perform) in advance of the second half, the majority of half-time practices represent very low exercise intensity, and are primarily passive in nature [46].

The commercially available sports beverages that are typically consumed pre- and during-competition (including at half-time), generally contain carbohydrates in 6–10% concentrations; of which high GI carbohydrate sources, such as glucose and maltodextrin, are common constituents. Paradoxically, high GI carbohydrates consumed within an hour of commencing a single bout of exercise may elicit rebound hypoglycemia 15–30 min into subsequent activity [47]. To explain the significance of such findings to soccer, consideration should be given to the differing physiological responses when carbohydrates are consumed during exercise, such as a high-intensity warm-up or throughout a match, versus those observed following consumption in a non-exercising state [48], such as at half-time.

Briefly, carbohydrates consumed at rest promote initial elevations in blood glucose concentrations. The subsequent actions of insulin attempt to normalize glycemia via a myriad of responses including a reduction in lipolysis, and increasing the liver, skeletal muscle and fat cell uptake of glucose [48,49]. Conversely, hyperglycemic responses are observed when exogenous carbohydrates are consumed during high-intensity exercise due to the actions of catecholamines, cortisol and growth hormone [48]. An additional consideration for half-time, is that insulin-independent glucose uptake occurs following a bout of prior exercise due to translocation of type four glucose transporters (GLUT4) [48,50]. Carbohydrate supplementation, and the glycemic response to high-intensity exercise undertaken following a period of recovery from an initial bout of exercise, has received little attention to date. This is somewhat surprising given the divergent nature of the physiological responses to carbohydrate consumed at rest and during exercise, the intermittent nature of soccer match-play, and the acknowledged importance of blood glucose concentrations for augmenting soccer-specific performance [18,25,26,39].

The common notion that exogenous carbohydrate consumption before and during intermittent exercise maintains glycemia throughout the duration of match-play has recently been challenged. Notably, when blood samples are taken frequently throughout each half, transient changes in blood glucose concentrations have been reported during soccer-specific exercise; particularly at the onset of the second half [26,33,39,43]. As previously mentioned, ingesting a 6% sucrose–electrolyte drink before and during (every 15 min) a simulated soccer match, attenuated reductions in post-exercise shooting performance [26]. However, this pattern of exogenous carbohydrate provision, including consumption throughout a passive half-time period, produced blood glucose concentrations that fell by 30% in the initial stages of the second half.

This transient lowering of blood glucose concentrations, termed the ‘exercise-induced rebound glycemic response’, has since been confirmed [33]. Interestingly, in a soccer match simulation that included a 15 min passive half-time period, >50% of participants reported hypoglycemic values (defined as blood glucose ≤3.8 mmol·L^−1^) at 60 min (i.e., 15 min into the second half) when carbohydrates were consumed before and during exercise [33]. Although the mechanisms responsible for, and the effects of, rapid reductions in blood glucose concentrations are currently unclear, optimization of match-day nutritional strategies is likely desirable for soccer players [46]. Adopting the premise of “assess then address” may provide a framework for practitioners and applied researchers to, (1) observe/audit current practices and thus establish whether key principles perceived to occur actually do so (e.g., are current carbohydrate ingestion strategies actually maintaining blood glucose concentrations for the full duration of match-play?), and (2) rationalise specific interventions (e.g., modify match-day carbohydrate intake as per the discussions below). The following discussion espouses potential modifications to current half-time practices by considering: (1) The GI of the carbohydrate consumed; (2) the timing of ingestion; (3) the amount/concentration of energy consumed; and (4) ingesting carbohydrate during a half-time rewarm-up.

### 3.2. Changing the Glycemic Index of Carbohydrate Consumed

Evidence, primarily from cycling studies, suggests that the GI of ingested carbohydrate may influence the rebound hypoglycemia observed at the onset of a single bout of exercise [51]; possibly due to the varying degrees of hyperinsulinemia observed post-ingestion. For example, when consumed 45 min prior to commencing activity, ingesting 75 g of trehalose (GI: 67) and galactose (GI: 20), which both exhibit a lower GI than glucose, attenuated the plasma insulin response and reduced the prevalence of hypoglycemia experienced during cycling exercise [51]. Thus, rebound hypoglycemia was substantially reduced when low to moderate GI (20–70) carbohydrates were ingested prior to a single bout of exercise, compared with high GI glucose (GI: 100). 

The paucity of studies investigating the effects of consuming low GI carbohydrates in soccer, may be attributed to concerns over the risk of gastric distress associated with products offering prolonged versus expedited appearance of exogenous energy during high-intensity exercise [52]. However, work by Stevenson et al. [53] investigated the effects of an 8% solution of low GI isomaltulose (GI: 32) consumed during the warm-up and at half-time throughout a soccer match simulation that included extra-time. Isomaltulose promotes a lower insulinaemic response and slower delivery of glucose into the systemic circulation when compared to higher GI sources of carbohydrate, and participants better maintained glycemia throughout the second half of exercise than when equivalent volumes of maltodextrin were consumed (GI: 90–100). Moreover, the dampened epinephrine response in the isomaltulose condition suggests a potential sparing of muscle glycogen; depletion of which may substantially contribute to the development of peripheral fatigue during soccer [11]. Although reducing the GI of the carbohydrate consumed did not attenuate declines in either physical or skilled performance, this may be explained by the low rate of overall consumption (~20 g·h^−1^). Crucially, abdominal comfort was not influenced by GI, therefore when limited opportunities for feeding exist (i.e., during soccer match-play), consuming low GI carbohydrates may represent an alternative to traditional forms of exogenous energy provision. 

### 3.3. Changing the Timing of Carbohydrate Ingestion

The temporal proximity at which carbohydrates are consumed prior to a single bout of activity can influence the metabolic responses to exercise. For example, Moseley et al. [47] investigated the effects of 75 g of glucose ingested 15, 45 or 75 min before commencing cycling. Plasma glucose and insulin concentrations were significantly elevated immediately before exercise when carbohydrate had been consumed 15 min prior to the onset of activity, whereas the lowest insulin concentrations were observed when 75 min separated carbohydrate ingestion and the onset of exercise. Additionally, consuming carbohydrate–electrolyte gels (providing 0.7 ± 0.1 g·kg^−1^ BM carbohydrates) following 90 min of exercise and ~5 min prior to a simulated extra-time period, enhanced dribbling performance and elevated blood glucose concentrations by ~16% in extra-time [25]. While studies specific to the soccer half-time period are lacking, the timing of carbohydrate feeding in proximity to the start of the second half has the potential to influence subsequent responses; especially those associated with blood glucose homeostasis. It may be that ingesting carbohydrates as close to the onset of subsequent activity (i.e., the final 5 min of half-time) produces similar physiological responses to those observed when carbohydrate is consumed during high-intensity exercise, and may attenuate the decline in glycemia currently observed when exogenous energy is consumed throughout passive half-time periods [26,33,39,43]. Although the short-duration of half-time may limit the extent to which modification of nutrient timing is possible, no studies have systematically examined the physiological or performance effects of providing carbohydrates at the start, middle or end of the half-time period. 

### 3.4. Changing the Amount/Concentration of Carbohydrate Consumed

In studies that have employed continuous exercise protocols and focused on water absorption as a priority, the detrimental effects on gastric emptying and intestinal absorption associated with high-concentration carbohydrate solutions, have led to recommendations that beverages containing between 5–10% carbohydrates are consumed during exercise [38,54]. However, limited data exists about the effects of providing carbohydrates in greater amounts (>10% solutions) when intermittent, as opposed to continuous, exercise is performed. This is somewhat surprising, given that ingesting a 20% glucose solution has been reported to enhance sprint capacity after 90 min (two 45 min periods, separated by 15 min) of intermittent cycling [55].

In recreational soccer players, elevated blood glucose concentrations have been observed from 75 min onwards when a 10% carbohydrate–electrolyte beverage was consumed before and during (including at half-time) a simulated soccer match, compared with a fluid-electrolyte placebo [33]. Interestingly, despite similarities in blood glucose concentrations (~4.0 mmol∙L^−1^) at 60 min, differences in glycemic responses were observed between conditions at 90 min. As pre-exercise ingestion of carbohydrates appear to elicit similar glycemic responses irrespective of dosage [56,57], and that the rebound hypoglycemic response appears to decay within the initial stages of exercise when high GI sources are consumed [47,55], provision of additional carbohydrate at half-time may plausibly afford ergogenic effects during the latter stages of a match. Indeed, dribbling and self-paced exercise performance were enhanced when 250 mL boluses of a 12% carbohydrate solution were consumed prior to each half of soccer specific exercise [43]. 

### 3.5. Consuming Carbohydrate during a Half-Time Rewarm-Up

It is well established that the combination of exogenous carbohydrate ingestion and high-intensity activity can elicit a hyperglycemic response in both clinical and non-clinical populations. As pancreatic beta-cell activity is inhibited by an exercise-induced catecholamine release [48], carbohydrates consumed during exercise can promote elevated blood glucose concentrations. Whilst practitioners often experience limited opportunities to engage in re-warm up activities at half-time [58], it is plausible that by combining high-intensity exercise and simultaneous carbohydrate ingestion during the half-time period, blood glucose concentrations could be better maintained thereafter.

In support, Brouns et al. [59] observed increased catecholamine concentrations, a blunted insulin response, and an increase in blood glucose concentrations at the onset of exercise, when 600 mL of a concentrated maltodextrin drink was consumed during the preceding 20 min cycle warm-up that included isolated sprint bouts. Although reductions in blood glucose concentrations were observed after 20 min of the continuous exercise that followed, these declines did not reach statistical significance. Moreover, whilst no effects on performance were observed, elevated blood glucose concentrations were reported in a subsequent exercise bout when a 16% solution containing 80 g sucrose was consumed during a previous 15 min bout of active recovery that followed a 4 km cycling time trial [60]. Although this active recovery was conducted at low intensity (i.e., <150 W), the authors speculated that the very high-intensity exercise (i.e., 4 km time trial) performed immediately before carbohydrate ingestion may have elevated blood glucose concentrations at the onset of subsequent exercise. Consequently, a half-time rewarm-up that includes a high-intensity component, combined with the ingestion of carbohydrates, may prove beneficial for soccer players who experience an exercise-induced rebound glycemic response at the onset of the second half [39]. However, the application of this approach is yet to be determined in situations where carbohydrates are provided during recovery from previous intermittent activity (i.e., first half), and when the subsequent exercise is also intermittent in nature (i.e., soccer match-play).

## 4. Summary

Consumption of carbohydrates both before and during competition is a nutritional strategy commonly recommended to soccer players. Such practices aim to preserve endogenous energy stores (including blood glucose concentrations and muscle glycogen), and attenuate declines in physical and skilled performance throughout the duration of match-play. Perturbations in blood glucose concentrations have been found to influence the quality of both cognitive and physical performances during soccer-specific exercise. Therefore, a role appears to exist for exogenous carbohydrate consumption to facilitate preservation of skilled performance under conditions of soccer-specific fatigue. Likewise, as evidence suggests that current practices may be sub-optimal, viable future research opportunities exist regarding strategies to maintain blood glucose concentrations for the full duration of a match. In particular, the half-time period poses unique opportunities for practitioners working in team sports such as soccer. Notably, transient reductions in blood glucose concentrations during the early stages of the second half have been observed when a variety of feeding patterns have been implemented. With these issues still unresolved, possible modifications to current match-day strategies which hold a plausible physiological rationale, include: changing the GI of the carbohydrate consumed, modifying the timing of carbohydrate provision within the half-time period, amending the dosage of carbohydrates consumed by players, and combining carbohydrate provision with a half-time rewarm-up. With this in mind, future research should seek to optimize the ergogenic potential of carbohydrate supplementation for soccer players; research which may provide commercially lucrative opportunities.
